# Supplementary material for: Doublons, topology and interactions in a one-dimensional lattice
Source: Sci Rep. 2021 Jun 15;11:12540. doi: 10.1038/s41598-021-91778-z (PMC8206211; doi:10.1038/s41598-021-91778-z)
Supplement: Supplementary file 1 — Supplementary files [file 41598_2021_91778_MOESM1_ESM.pdf]

Supporting Information:
Doublons, topology and interactions in a one-dimensional lattice

P. Martínez Azcona[1] and C. A. Downing[2, *]

[1]*Departamento de Física de la Materia Condensada, Universidad de Zaragoza, Zaragoza 50009, Spain*
[2]*Department of Physics and Astronomy, University of Exeter, Exeter EX4 4QL, United Kingdom*

In this Supporting Information, we present the background theory supporting the results reported in the main text. Gradually building up to the model of the main text, we survey the anharmonic oscillator, the Bose-Hubbard dimer, the Bose-Hubbard chain, and the Bose-Hubbard dimerized chain. We study both the one and two excitation subspaces of each model, and we pay particular attention to any topological aspects.

CONTENTS

I. Anharmonic oscillator 2

II. Bose-Hubbard dimer 2
- A. One excitation subspace 3
  - 1. Entanglement 3
- B. Two excitation subspace 4
  - 1. Strong repulsive interactions 5
  - 2. Strong attractive interactions 6
  - 3. Weak interactions 6
  - 4. Entanglement 6

III. Bose-Hubbard chain 7
- A. One excitation subspace 8
  - 1. Infinite chain 8
  - 2. Finite chain 8
  - 3. Entanglement 9
- B. Two excitation subspace 9
  - 1. Scattering states 10
  - 2. Bound states 11
  - 3. Numerical diagonalization 12
  - 4. Entanglement 13

IV. Bose-Hubbard dimerized chain 14
- A. One excitation subspace 15
  - 1. Infinite chain 15
  - 2. Finite chain 17
  - 3. Entanglement 17
- B. Two excitation subspace 18

References 19

* c.a.downing@exeter.ac.uk

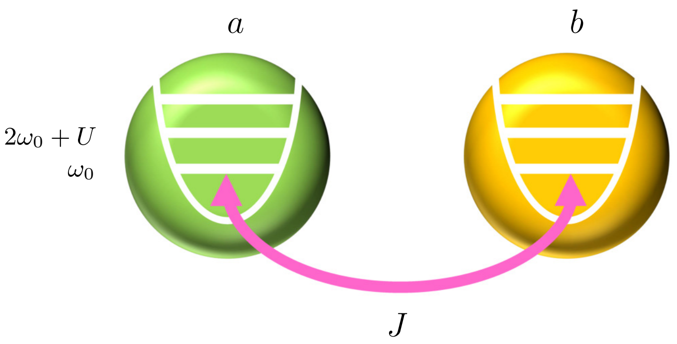

FIG. S1. A sketch of the Bose-Hubbard dimer, where each oscillator (labelled a and b) is of resonance frequency ω_0, and the on-site interaction U may be repulsive ($U > 0$) or attractive ($U < 0$). The oscillators are coupled via with the attractive coupling constant $-J < 0$.

I. ANHARMONIC OSCILLATOR

The building block of the dimerized chain Hamiltonian, given as Eq. (1) in the main text, is that of an anharmonic oscillator. Here we briefly recap the basics of a single anharmonic oscillator. The relevant Hamiltonian operator $\hat{H}$ reads

$$\hat{H} = \omega_0 b^\dagger b + \frac{U}{2} b^\dagger b^\dagger b b, \tag{S1}$$

describing an oscillator of resonance frequency ω_0 and anharmonicity U. The annihilation (creation) operator $b^\dagger$ (b) creates (destroys) an excitation on the oscillator. The eigenfrequency $\omega^{(\mathcal{N})}$ of the oscillator with $\mathcal{N}$ excitations reads

$$\omega^{(\mathcal{N})} = \mathcal{N}\omega_0 + \frac{\mathcal{N}}{2}(\mathcal{N}-1)\,U. \tag{S2}$$

Therefore, in the $\mathcal{N} = \{1, 2\}$ excitation sectors that we are interested in throughout this work, we see that the single excitation eigenfrequency $\omega^{(1)} = \omega_0$ is of course independent of the interaction strength U, while the doubly excited state is associated with the eigenfrequency $\omega^{(2)} = 2\omega_0 + U$, and thus carries the extra energy cost U due to the interaction.

II. BOSE-HUBBARD DIMER

In this section, we describe in detail the theory of a Bose-Hubbard dimer. The model is equivalent to the limiting case of $N = 2$ in the dimerized chain model given as Eq. (1) in the main text, and due to its simplicity it allows for insight into short chains. The Hamiltonian operator $\hat{H}$ describing a pair of coupled oscillators (each of resonance frequency ω_0) reads

$$\hat{H} = \omega_0\left(a^\dagger a + b^\dagger b\right) - J\left(a^\dagger b + b^\dagger a\right) + \frac{U}{2}\left(a^\dagger a^\dagger a a + b^\dagger b^\dagger b b\right), \tag{S3}$$

where $-J < 0$ is the attractive coupling constant, and the on-site interaction $U > 0$ ($U < 0$) is repulsive (attractive). The creation (annihilation) operators $a^\dagger$ and $b^\dagger$ (a and b) create (destroy) an excitation on the first and second oscillator respectively. The dimer system is sketched in Fig. S1, where the green (yellow) oscillator is of the a (b) type. In the basis of zero, one and two excitations, that is $\mathbf{0} = \{|0,0\rangle\}$, $\mathbf{1} = \{|1,0\rangle, |0,1\rangle\}$ and $\mathbf{2} = \{||2,0\rangle\rangle, ||1,1\rangle\rangle, ||0,2\rangle\rangle\}$, Eq. (S26) leads to the block diagonal matrix

$$H = \begin{pmatrix} \mathcal{H}_0 & 0 & 0 \\ 0 & \mathcal{H}_1 & 0 \\ 0 & 0 & \mathcal{H}_2 \end{pmatrix}, \tag{S4}$$

where $\mathcal{H}_\mathcal{N}$ spans the subspace with $\mathcal{N} = \{0, 1, 2\}$ excitations. We use $||i\rangle\rangle$ to refer to an eigenstate in the two excitation sector ($\mathcal{N} = 2$), as opposed to $|i\rangle$ for the one excitation sector ($\mathcal{N} = 1$). The trivial Hamiltonian $\mathcal{H}_0$ is associated with the ground eigenfrequency $\omega^{(0)} = 0$, and the vacuum eigenstate $|0,0\rangle$. In what follows in Sec. II A and Sec. II B, we examine the one and two excitation subspaces as described by $\mathcal{H}_1$ and $\mathcal{H}_2$ respectively. In particular we focus on their eigenfrequencies $\omega^{(\mathcal{N})}$, their eigenstates, and their entanglement properties.

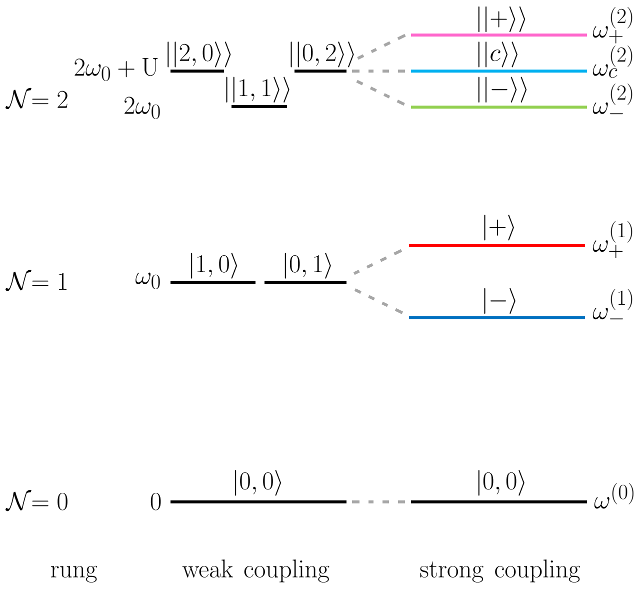

FIG. S2. Energy ladder of the Bose-Hubbard dimer, in the weak (left) and strong (right) coupling regimes [cf. Eq. (S6) and Eq. (S13)]. We restrict the ladder to the 0th, 1st and 2nd rungs, which are associated with $\mathcal{N} = \{0, 1, 2\}$ excitations.

A. One excitation subspace

The one excitation subspace $\mathcal{H}_1$, written in the basis $\{|1,0\rangle, |0,1\rangle\}$ and introduced in Eq. (S4), simply reads

$$\mathcal{H}_1 = \begin{pmatrix} \omega_0 & -J \\ -J & \omega_0 \end{pmatrix}, \tag{S5}$$

such that the two eigenfrequencies $\omega_i^{(1)}$ are

$$\omega_\pm^{(1)} = \omega_0 \pm J, \tag{S6}$$

which display a splitting of $2J$ between the upper and lower energy levels, which are labelled with the index $\pm$. The associated eigenstates $|\pm\rangle$ are symmetric and antisymmetric combinations of the uncoupled states,

$$|\pm\rangle = \frac{1}{\sqrt{2}} \left(|1,0\rangle \mp |0,1\rangle \right). \tag{S7}$$

These basic features can be seen in the first ($\mathcal{N} = 1$) rung of the energy ladder of the system, as sketched in the weak and strong coupling regimes in the left- and right-hand sides respectively of Fig. S2. The antisymmetric ($\uparrow\downarrow$) state $|+\rangle$ is associated with the upper frequency $\omega_+^{(1)}$ due to the attractiveness of the oscillator-oscillator coupling ($-J < 0$), while the symmetric ($\uparrow\uparrow$) state $|-\rangle$ is linked to the lower frequency $\omega_-^{(1)}$.

1. *Entanglement*

We are interested in two quantities which measure the degree of mixedness of the states of the system [1, 2]. Firstly, the purity ζ of the density matrix, as defined by

$$\zeta = \mathrm{Tr}\left(\rho^2\right), \tag{S8}$$

such that pure states are associated with $\zeta = 1$ and mixed states are associated with $\zeta < 1$. Maximally mixed states are defined by $\zeta = 1/D$, where D is the dimension of the Hilbert space. Secondly, we consider the Von Neumann entropy S, which is given by

$$S = -\mathrm{Tr}\{\rho \ln(\rho)\} = -\sum_n \lambda_n \ln(\lambda_n), \tag{S9}$$

where λ_n are the eigenvalues of the density matrix ρ. For a pure density matrix, one eigenvalue is unity and the rest are zero, so that $S = 0$. The opposing limiting case of a maximally mixed density matrix is characterized by $S = \ln(D)$, since all eigenvalues $\lambda_n = 1/D$ in the D-dimensional Hilbert space. Otherwise, mixed states have an entropy S in between these bounds, which quantifies the amount of information associated with each state in the system.

One may find the entanglement matrix M of a state by partitioning the system evenly (cutting it through the central point), and associating the rows and columns of M with states in the left and right hand sides of the partition [3, 4]. For the case of the dimer in the single excitation sector, as described by Eq. (S11), the entanglement matrices $M_\pm$ associated with the states $|\pm\rangle$ of Eq. (S7) arise from partitioning the dimer into one oscillator on the left (green sphere in Fig. S1) and one on the right (orange sphere). Let us make the columns of $M_\pm$ be associated with the right oscillator states $|\cdot 0\rangle$ and $|\cdot 1\rangle$, and the rows with the left oscillator states $|0\cdot\rangle$ and $|1\cdot\rangle$, so that

$$M_\pm = \frac{1}{\sqrt{2}} \begin{pmatrix} 0 & 1 \\ \mp 1 & 0 \end{pmatrix}. \tag{S10}$$

The connection to the reduced density matrices $\rho_\pm$ is given by [3, 4]

$$\rho_\pm = M_\pm^\dagger M_\pm = \frac{1}{2} \begin{pmatrix} 1 & 0 \\ 0 & 1 \end{pmatrix}. \tag{S11}$$

We directly find the purities $\zeta_\pm = 1/2$ from Eq. (S8) and the entropies $S_\pm = \ln(2) \simeq 0.693$ from Eq. (S9). These results are unsurprising since we are dealing with maximally mixed states in a $D = 2$ dimensional system, as is apparent from Eq. (S7). However, these measures become increasingly important in more complicated systems, as we shall see.

B. Two excitation subspace

The two excitation subspace $\mathcal{H}_2$, introduced in Eq. (S4), is three-dimensional and is given by

$$\mathcal{H}_2 = \begin{pmatrix} 2\omega_0 + U & -\sqrt{2}J & 0 \\ -\sqrt{2}J & 2\omega_0 & -\sqrt{2}J \\ 0 & -\sqrt{2}J & 2\omega_0 + U \end{pmatrix}, \tag{S12}$$

in the basis $\{||2,0\rangle\rangle, ||1,1\rangle\rangle, ||0,2\rangle\rangle\}$. The three eigenfrequencies $\omega_i^{(2)}$ are given by

$$\omega_+^{(2)} = 2\omega_0 + \frac{U}{2} + \sqrt{(2J)^2 + \left(\frac{U}{2}\right)^2}, \tag{S13a}$$

$$\omega_c^{(2)} = 2\omega_0 + U, \tag{S13b}$$

$$\omega_-^{(2)} = 2\omega_0 + \frac{U}{2} - \sqrt{(2J)^2 + \left(\frac{U}{2}\right)^2}, \tag{S13c}$$

where the index refers to the upper $(+)$, central (c) and lower $(-)$ energy levels. The associated eigenstates read

$$||+\rangle\rangle = \frac{1}{\sqrt{2 + \frac{1}{2}\left(\frac{\omega_-^{(2)} - 2\omega_0}{J}\right)^2}} \begin{pmatrix} 1 \\ \frac{\omega_-^{(2)} - 2\omega_0}{\sqrt{2}J} \\ 1 \end{pmatrix}, \tag{S14a}$$

$$||c\rangle\rangle = \frac{1}{\sqrt{2}} \begin{pmatrix} 1 \\ 0 \\ -1 \end{pmatrix}, \tag{S14b}$$

$$||-\rangle\rangle = \frac{1}{\sqrt{2 + \frac{1}{2}\left(\frac{\omega_+^{(2)} - 2\omega_0}{J}\right)^2}} \begin{pmatrix} 1 \\ \frac{\omega_+^{(2)} - 2\omega_0}{\sqrt{2}J} \\ 1 \end{pmatrix}, \tag{S14c}$$

again in the basis $\{||2,0\rangle\rangle, ||1,1\rangle\rangle, ||0,2\rangle\rangle\}$. These results reveal two states, $||+\rangle\rangle$ and $||-\rangle\rangle$, which are in general associated with the whole set of bare eigenstates $||2,0\rangle\rangle$, $||1,1\rangle\rangle$, and $||0,2\rangle\rangle$. The other state $||c\rangle\rangle$ is a doublon: it arises wholly from the

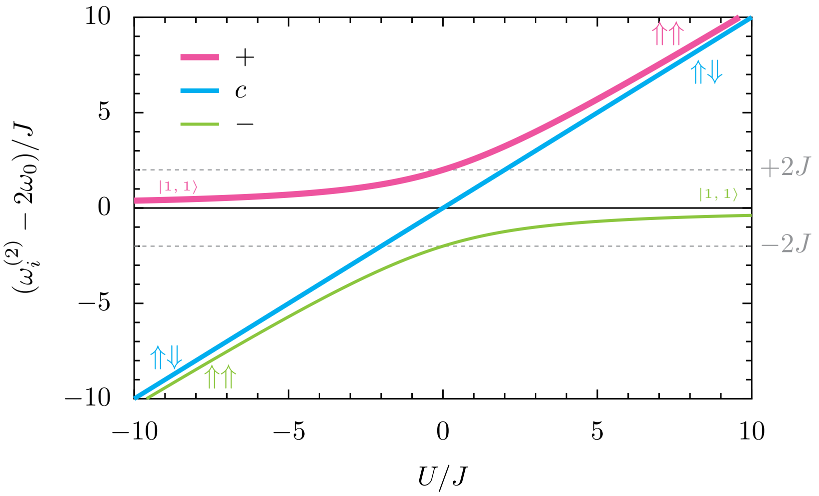

FIG. S3. Dimer eigenfrequencies $\omega_i^{(2)}$ in the two excitation sector (as measured from $2\omega_0$) as a function of the interaction strength U, in units of the coupling strength J [cf. Eq. (S13)]. Dashed lines: guides for the eye at $\pm 2J$. Labels: symmetric and antisymmetric asymptotics are represented by ⇑⇑ and ⇑⇓ respectively.

doubly-occupied states $||2,0\rangle\rangle$ and $||0,2\rangle\rangle$, and has an eigenfrequency $\omega_c^{(2)}$ which is independent of the coupling constant J. We plot in Fig. S3 the eigenfrequencies $\omega_i^{(2)}$ as a function of the interaction strength U, in units of the coupling strength J, using Eq. (S13). The dashed lines are guides for the eye at $\pm 2J$, while the labels near to the colored lines describe the asymptotic behavior of the eigenstate, which can be symmetric like $\left(||20\rangle\rangle + ||02\rangle\rangle\right)/\sqrt{2}$ as represented by ⇑⇑, or antisymmetric like $\left(||20\rangle\rangle - ||02\rangle\rangle\right)/\sqrt{2}$ as represented by ⇑⇓. In what follows, we explore the limiting cases of strong repulsive interactions, strong attractive interactions, and weak interactions, to fully characterize the behavior shown in Fig. S3.

1. *Strong repulsive interactions*

Let us consider the limit of strong repulsive interactions ($U \to +\infty$). The eigenfrequencies $\omega_i^{(2)}$ of Eq. (S13) tend towards

$$\lim_{U\to+\infty} \omega_+^{(2)} = 2\omega_0 + U, \tag{S15a}$$

$$\lim_{U\to+\infty} \omega_c^{(2)} = 2\omega_0 + U, \tag{S15b}$$

$$\lim_{U\to+\infty} \omega_-^{(2)} = 2\omega_0, \tag{S15c}$$

and the eigenstates of Eq. (S14) become

$$\lim_{U\to+\infty} ||+\rangle\rangle = \frac{1}{\sqrt{2}}\left(||20\rangle\rangle + ||02\rangle\rangle\right), \tag{S16a}$$

$$\lim_{U\to+\infty} ||c\rangle\rangle = \frac{1}{\sqrt{2}}\left(||20\rangle\rangle - ||02\rangle\rangle\right), \tag{S16b}$$

$$\lim_{U\to+\infty} ||-\rangle\rangle = ||11\rangle\rangle. \tag{S16c}$$

This behavior of the eigenfrequencies is displayed in Fig. S3, where the superposition of doubly-occupied states associated with $\omega_+^{(2)}$ and $\omega_c^{(2)}$ both scale linearly with U. The highest energy state $||+\rangle\rangle$ is symmetric (⇑⇑) and the next highest energy state $||c\rangle\rangle$ is antisymmetric (⇑⇓). The twice singly occupied state $||-\rangle\rangle$, associated with $\omega_-^{(2)}$, is U independent and effectively uncoupled.

2. *Strong attractive interactions*

The limit of strong attractive interactions ($U \to -\infty$) shows a similar behavior. The eigenfrequencies $\omega_i^{(2)}$ of Eq. (S13) approach

$$\lim_{U\to-\infty} \omega_+^{(2)} = 2\omega_0, \tag{S17a}$$

$$\lim_{U\to-\infty} \omega_c^{(2)} = 2\omega_0 + U, \tag{S17b}$$

$$\lim_{U\to-\infty} \omega_-^{(2)} = 2\omega_0 + U, \tag{S17c}$$

and the linked eigenstates of Eq. (S14) tend towards

$$\lim_{U\to-\infty} ||+\rangle\rangle = ||11\rangle\rangle, \tag{S18a}$$

$$\lim_{U\to-\infty} ||c\rangle\rangle = \frac{1}{\sqrt{2}}\left(||20\rangle\rangle - ||02\rangle\rangle\right), \tag{S18b}$$

$$\lim_{U\to-\infty} ||-\rangle\rangle = \frac{1}{\sqrt{2}}\left(||20\rangle\rangle + ||02\rangle\rangle\right), \tag{S18c}$$

showing again that the superposition of doubly-occupied states, now associated with $\omega_c^{(2)}$ and $\omega_-^{(2)}$, scale linearly with U. Here the lowest energy state $||-\rangle\rangle$ is symmetric ($\Uparrow\Uparrow$), and the next highest energy state $||c\rangle\rangle$ is antisymmetric ($\Uparrow\Downarrow$), as shown in Fig. S3. The twice singly occupied state $||+\rangle\rangle$ is now associated with $\omega_+^{(2)}$, and is U independent and essentially uncoupled.

3. *Weak interactions*

Finally, we consider the limit of vanishing interactions ($U \to 0$). The eigenfrequencies $\omega_i^{(2)}$ of Eq. (S13) then approach

$$\lim_{U\to0} \omega_+^{(2)} = 2\omega_0 + 2J, \tag{S19a}$$

$$\lim_{U\to0} \omega_c^{(2)} = 2\omega_0, \tag{S19b}$$

$$\lim_{U\to0} \omega_-^{(2)} = 2\omega_0 - 2J, \tag{S19c}$$

as may be seen from Fig. S3 at the intersections of the colored lines and dashed horizontal lines. The associated eigenstates of Eq. (S14) tend towards the simple forms

$$\lim_{U\to0} ||+\rangle\rangle = \frac{1}{2}\left(||20\rangle\rangle - \sqrt{2}||11\rangle\rangle + ||02\rangle\rangle\right), \tag{S20a}$$

$$\lim_{U\to0} ||c\rangle\rangle = \frac{1}{\sqrt{2}}\left(||20\rangle\rangle - ||02\rangle\rangle\right), \tag{S20b}$$

$$\lim_{U\to0} ||-\rangle\rangle = \frac{1}{2}\left(||20\rangle\rangle + \sqrt{2}||11\rangle\rangle + ||02\rangle\rangle\right). \tag{S20c}$$

4. *Entanglement*

In the same fashion as for the one excitation sector [cf. Eq. (S10) and Eq. (S11)], the density matrices ρ_i of the three two-particle states $\{||+\rangle\rangle, ||c\rangle\rangle, ||-\rangle\rangle\}$ may be calculated via entanglement matrices, leading to

$$\rho_c = \frac{1}{2}\begin{pmatrix} 1 & 0 & 0 \\ 0 & 0 & 0 \\ 0 & 0 & 1 \end{pmatrix}, \quad \rho_\pm = \begin{pmatrix} \frac{1}{4}\left(1 \pm \frac{U}{\sqrt{16J^2+U^2}}\right) & 0 & 0 \\ 0 & \frac{1}{2}\left(1 \mp \frac{U}{\sqrt{16J^2+U^2}}\right) & 0 \\ 0 & 0 & \frac{1}{4}\left(1 \pm \frac{U}{\sqrt{16J^2+U^2}}\right) \end{pmatrix}, \tag{S21}$$

which of course satisfy $\mathrm{Tr}(\rho_i) = 1$. The purities of Eq. (S21) follow from Eq. (S8) as

$$\zeta_c = \frac{1}{2}, \quad \zeta_\pm = \frac{1}{8}\left(3 + \frac{3U^2}{16J^2+U^2} \mp \frac{2U}{\sqrt{16J^2+U^2}}\right). \tag{S22}$$

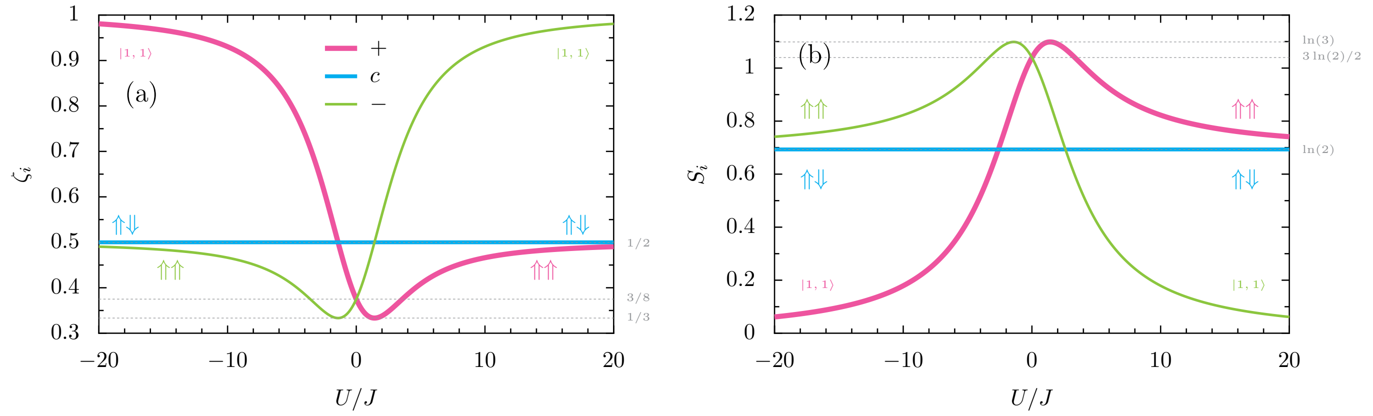

FIG. S4. Panel (a): purity ζ_i of the dimer states in the two excitation sector, as a function of the interaction strength U, in units of the coupling strength J [cf. Eq. (S22)]. Panel (b): the associated entropy S_i [cf. Eq. (S24)]. Dashed lines: guides for the eye at various points of interest.

Since the Hilbert space is $D = 3$ dimensional, the purities span the range $1/3 \le \zeta_i \le 1$, where the lower bound describes maximally mixed states and the upper bound pure states. While ζ_c is constant due to the U-independent form of the state, the U-dependent states have purities $\zeta_\pm$ which exhibit the following behaviors in critical limiting cases

$$\zeta_+ \to \begin{cases} 1/2, & U \to +\infty \\ 3/8, & U \to 0, \\ 1, & U \to -\infty, \end{cases} \qquad \zeta_- \to \begin{cases} 1, & U \to +\infty \\ 3/8, & U \to 0, \\ 1/2, & U \to -\infty. \end{cases} \tag{S23}$$

The global minimum is $\min\{\zeta_\pm\} = 1/3$, which occurs at $U = \sqrt{2}J$ for ζ_+ and $U = -\sqrt{2}J$ for ζ_-, coinciding with the point at which the relevant eigenstate becomes maximally mixed, with a form like $(1, -1, 1)/\sqrt{3}$. We plot the purities of Eq. (S22) in Fig. S4 (a), as a function of the interaction strength U, showing the aforementioned features. Likewise, the Von Neumann entropies satisfy $0 \le S_i \le \ln(3)$ and follow from Eq. (S9) as

$$S_c = \ln(2), \quad S_\pm = \frac{1}{2}\left\{ \ln\left(8 + \frac{U^2}{2J^2}\right) \pm \frac{U}{\sqrt{16J^2 + U^2}} \ln\left(2 + \frac{U^2 + U\sqrt{16J^2+U^2}}{4J^2}\right) \right\}, \tag{S24}$$

which contain the following limiting cases

$$S_+ \to \begin{cases} \ln(2), & U \to +\infty \\ \frac{3}{2}\ln(2), & U \to 0, \\ 0, & U \to -\infty, \end{cases} \qquad S_- \to \begin{cases} 0, & U \to +\infty \\ \frac{3}{2}\ln(2), & U \to 0, \\ \ln(2), & U \to -\infty, \end{cases} \tag{S25}$$

where $\ln(2) \simeq 0.693$ and $(3/2)\ln(2) \simeq 1.04$. The global maxima $\max\{S_\pm\} = \ln(3) \simeq 1.10$, which occurs at $U = \sqrt{2}J$ for S_+ and $U = -\sqrt{2}J$ for S_-, pinpoints the instances of maximally mixed eigenstates. We plot the entropies of Eq. (S24) in Fig. S4 (b), which shows the features just mentioned.

III. BOSE-HUBBARD CHAIN

In this section, we examine the theory of the Bose-Hubbard regular chain, a model which is recovered by the dimerized chain of Eq. (1) in the main text in the limit of $J_1 = J_2$. The Bose-Hubbard chain captures several features also apparent in the dimerized chain, such as the existence of bands of purely scattering states and purely bound states. Furthermore, the theoretical techniques which we use to treat the regular chain can also be generalized to use with the more complicated dimerized chain.

We consider a regularly spaced chain of N oscillators, each of resonance frequency ω_0 and separated by the distance d, as sketched in Fig. S5. The nearest-neighbor coupling constant $-J < 0$ is attractive, and the on-site interaction U may be repulsive ($U > 0$) or attractive ($U < 0$). The Hamiltonian operator $\hat{H}$ describing this system reads

$$\hat{H} = \omega_0 \sum_{n=1}^{N} b_n^\dagger b_n - J \sum_{n=1}^{N-1} \left(b_n^\dagger b_{n+1} + b_{n+1}^\dagger b_n \right) + \frac{U}{2} \sum_{n=1}^{N} b_n^\dagger b_n^\dagger b_n b_n, \tag{S26}$$

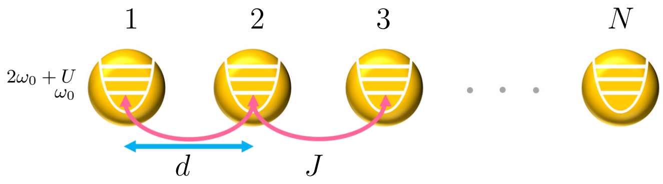

FIG. S5. A sketch of the Bose-Hubbard chain of N oscillators, each of resonance frequency ω_0 and separated by the distance d. The nearest-neighbor coupling constant $-J < 0$ is attractive, and the on-site interaction U may be repulsive ($U > 0$) or attractive ($U < 0$).

where the annihilation (creation) operator $b_n^\dagger$ (b_n) creates (destroys) an excitation on oscillator n. In the same way as for the Bose-Hubbard dimer of Appendix II, in what follows we consider the $\mathcal{N} = \{1, 2\}$ excitation subspaces of the system in Sec. III A and Sec. III B respectively.

A. One excitation subspace

In the one excitation subspace $\mathcal{H}_1$ there are of course no on-site interactions, and so the Hamiltonian of Eq. (S26) reduces to

$$\mathcal{H}_1 = \omega_0 \sum_{n=1}^{N} b_n^\dagger b_n - J \sum_{n=1}^{N-1} \left(b_n^\dagger b_{n+1} + b_{n+1}^\dagger b_n \right). \tag{S27}$$

We analyze this Hamiltonian in the infinite ($N \gg 1$) chain limit in Part III A 1, and for a general finite chain in Part III A 2.

1. *Infinite chain*

In the long chain limit of $N \gg 1$, we employ periodic boundary conditions for simplicity, such that we can utilize the Fourier transform

$$b_n = \frac{1}{\sqrt{N}} \sum_q \mathrm{e}^{inqd} b_q, \tag{S28}$$

where the quasi-momentum $q = 2\pi m/Nd$, where $m \in [-N/2, +N/2]$ is an integer. The momentum space creation (annihilation) operator $b_q^\dagger$ (b_q) creates (destroys) an excitation with wavenumber q. The transformation of Eq. (S28), along with Eq. (S27), immediately yields the diagonal form of the one excitation Hamiltonian

$$\mathcal{H}_1 = \sum_q \omega_q^{(1)} b_q^\dagger b_q, \tag{S29}$$

where the eigenfrequencies $\omega_q^{(1)}$ are given by

$$\omega_q^{(1)} = \omega_0 - 2J \cos(qd). \tag{S30}$$

This well-known spectrum is plotted within the first Brillouin zone as the solid red line in Fig. S6. Clearly, the one excitation spectrum is bounded by $-2J \leq \omega_k^{(1)} - \omega_0 \leq 2J$, and thus has the bandwidth

$$\mathcal{B}^{(1)} = 4J, \tag{S31}$$

as marked with dashed lines in Fig. S6.

2. *Finite chain*

When considering a finite chain of size N, we utilize the discrete sine transform

$$b_n = \frac{1}{\sqrt{N+1}} \sum_m \sin\left(\tfrac{n\pi m}{N+1}\right) b_m, \tag{S32}$$

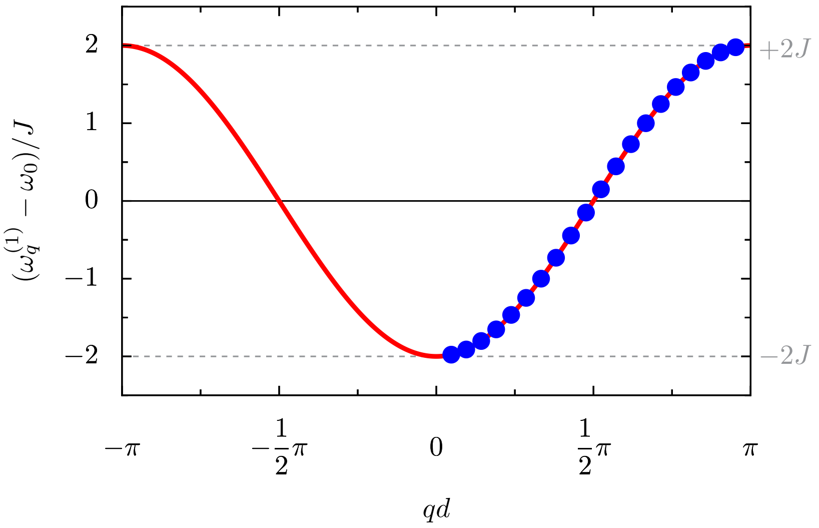

FIG. S6. Eigenfrequencies $\omega_q^{(1)}$ of an infinite chain in the one excitation sector (as measured from ω_0), in units of the coupling strength J, as a function of the wavevector q [cf. Eq. (S30)]. Blue circles: eigenfrequencies $\omega_m^{(1)}$ for a finite chain of $N = 20$ oscillators [cf. Eq. (S34)]. Dashed lines: guides for the eye at $\pm 2J$.

where the integer $m \in [1, N]$, and which satisfies the open boundary conditions $b_0 = b_{N+1} = 0$. Upon substituting the transformation of Eq. (S32) into Eq. (S27), we find the diagonalized Hamiltonian

$$\mathcal{H}_1 = \sum_m \omega_m^{(1)} b_m^\dagger b_m, \tag{S33}$$

where the finite chain eigenfrequencies $\omega_m^{(1)}$ read [cf. Eq. (S30)]

$$\omega_m^{(1)} = \omega_0 - 2J \cos\left(\frac{\pi m}{N+1}\right). \tag{S34}$$

In the long chain limit $N \gg 1$, Eq. (S34) recovers the infinite chain result of Eq. (S30) (for $q > 0$ only, because of the different boundary conditions employed in the two cases). We may assign the quasi-momentum q in the finite chain case via the relation $\pi m/(N+1) \to qd$. We plot Eq. (S34) as the blue circles in Fig. S6 for a chain of size $N = 20$, showing the excellent agreement with the infinite chain result (represented by the solid red line).

3. *Entanglement*

The purity ζ_m of the eigenstates of the finite chain can be calculated from Eq. (S8) for some chain of even size N, and the results are plotted in Fig. S7. The dimer ($N = 2$) and infinite chain ($N \to \infty$) limits are given by

$$\zeta_m \to \begin{cases} 1/2, & N = 2, \\ 1/4, & N \to \infty, \end{cases} \tag{S35}$$

as can be seen from Fig. S7. The results are the same for all states, that is of any m, since all states are equally extended throughout the chain and the asymptotic limit of $\zeta_m = 1/4$ is quickly reached, due to the nature of the eigenstates as superpositions of sine waves.

B. Two excitation subspace

In the two excitation sector, we apply the two-particle eigenstate

$$|\psi\rangle = \sum_{m,n=1}^{N} c_{m,n}\, b_m^\dagger b_n^\dagger |\text{vac}\rangle, \tag{S36}$$

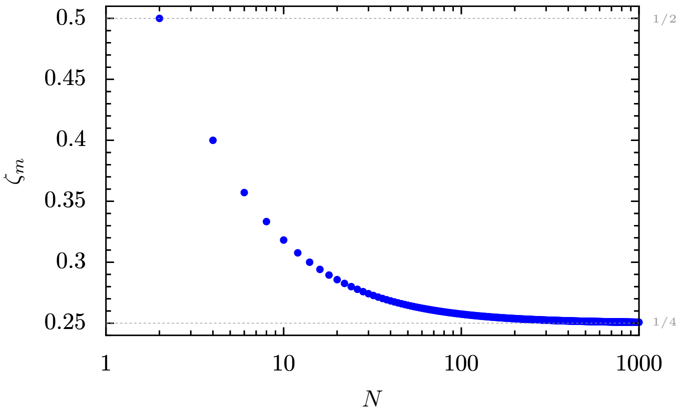

FIG. S7. Purity ζ_m of the chain states (labelled by m) in the one excitation sector, as a function of the (even) number of oscillators in the chain N. Dashed lines: guides for the eye at the upper and lower bounds of the purity [cf. Eq. (S35)].

to the Schrödinger equation $\hat{H}|\psi\rangle = \omega^{(2)}|\psi\rangle$, where $\hat{H}$ is given by Eq. (S26) and where $\omega^{(2)}$ are the two-particle eigenvalues. This procedure leads to the recurrence equation

$$2\omega_0 c_{n,m} - J\left(c_{n+1,m} + c_{n-1,m} + c_{n,m+1} + c_{n,m-1}\right) + U\delta_{n,m}c_{n,m} = \omega^{(2)}c_{n,m}, \tag{S37}$$

where $\delta_{i,j}$ is the Kronecker delta. As shown by Valiente and Petrosyan [5, 6], this eigenproblem can be solved exactly in the long chain limit using a Bethe ansatz. Let us try the following separable solution

$$c_{n,m} = \mathrm{e}^{\mathrm{i}\left(\frac{m+n}{2}\right)Kd} f(m-n), \tag{S38}$$

where $K \in [-\pi/d, +\pi/d]$ is a the wavevector associated with the center-of-mass coordinate. Substitution of Eq. (S38) into Eq. (S37) yields

$$-2J\cos\left(\tfrac{Kd}{2}\right)\Big[f(m-n+1) + f(m-n-1)\Big] + U\delta_{m-n,0}f(m-n) = \Big[\omega^{(2)} - 2\omega_0\Big]f(m-n), \tag{S39}$$

This recurrence equation admits solutions corresponding to both scattering states and bound states, which we consider each in turn in Part III B 1 and Part III B 2 respectively.

1. *Scattering states*

Scattering states are extended throughout the chain and thus can be well described with the trigonometric ansatz

$$f(m-n) = A\cos\left([m-n]kd\right) + B\sin\left(|m-n|kd\right), \tag{S40}$$

where k is the wavenumber associated with the relative coordinate, and the weighting coefficients A and B are unknowns to be found. Inserting Eq. (S40), which obeys the required bosonic symmetry $f(m-n) = f(n-m)$, into Eq. (S39) leads to the eigenfrequencies $\omega_{\mathrm{S}}^{(2)}$ of the scattering band

$$\omega_{\mathrm{S}}^{(2)} = 2\omega_0 - 4J\cos\left(\tfrac{Kd}{2}\right)\cos\left(kd\right). \tag{S41}$$

The value of the coefficient B follows as

$$B = \frac{U}{4J\sin\left(kd\right)\cos\left(\frac{Kd}{2}\right)}A, \tag{S42}$$

where A is fixed by the normalization. This result reveals that for vanishing ($U \to 0$) interactions Eq. (S40) behaves as

$$\lim_{U\to 0} f(m-n) \propto \cos\left([m-n]kd\right), \tag{S43}$$

recovering the solution of non-interacting bosons. However, for strong ($U \to \pm\infty$) interactions we find

$$\lim_{U\to\pm\infty} f(m-n) \propto \sin\left(|m-n|kd\right), \tag{S44}$$

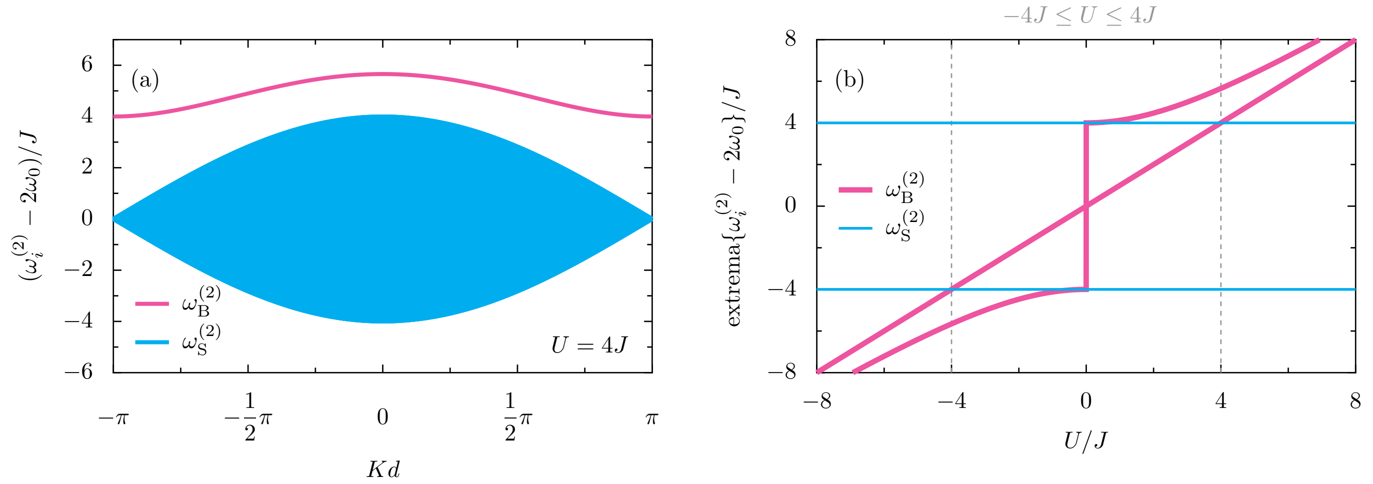

FIG. S8. Panel (a): eigenfrequencies $\omega_i^{(2)}$ of an infinite chain in the two excitation sector (as measured from $2\omega_0$), in units of the coupling strength J, as a function of the center-of-mass wavevector K. The band of bound states $\omega_B^{(2)}$ is shown by the pink line [cf. Eq. (S50)], and the bands of scattering states $\omega_S^{(2)}$ are shown by the cyan region [cf. Eq. (S41)]. In the panel, $U = 4J$. Panel (b): the extrema (the maximum and minimum) of the eigenfrequencies $\omega_i^{(2)}$ as a function of the interaction strength U, in units of the coupling strength J. Vertical dashed lines: guides for the eye for the region $-4J \leq U \leq 4J$, where the bound state band merges with the scattering bands.

which displays a fermion-like result, since at $m = n$ we have $f(0) = 0$. The scattering state spectrum of Eq. (S41) is plotted in blue in Fig. S8 (a), which displays the dispersion leading to the scattering bandwidth

$$\mathcal{B}_S^{(2)} = 8J, \tag{S45}$$

as displayed in Fig. S8 (b), which is double that of the single excitation bandwidth $\mathcal{B}^{(1)}$ as given by Eq. (S31).

2. *Bound states*

The physics of the bound states are captured by the exponentially decaying trial solution

$$f(m-n) = C g^{|m-n|}, \tag{S46}$$

where g is an unknown to be found, and C is a normalization constant. Substitution of Eq. (S46) into Eq. (S39) leads to two distinct equations. Firstly, when $m \neq n$ we find the set of equations

$$-2J\cos\left(\tfrac{Kd}{2}\right)\left[g^{|m-n+1|} + g^{|m-n-1|}\right] = \left[\omega^{(2)} - 2\omega_0\right] g^{|m-n|}, \quad m \neq n, \tag{S47}$$

and secondly, when $m = n$ we have the boundary condition

$$-4J\cos\left(\tfrac{Kd}{2}\right) g = \omega^{(2)} - 2\omega_0 - U, \quad m = n. \tag{S48}$$

The above equations are solved when g satisfies

$$g = \frac{U}{4J\cos\left(\frac{Kd}{2}\right)} - \mathrm{sgn}\{U\}\sqrt{1 + \left(\frac{U}{4J\cos\left(\frac{Kd}{2}\right)}\right)^2}, \tag{S49}$$

which reveals the bound state spectrum to be

$$\omega_B^{(2)} = 2\omega_0 + \mathrm{sgn}\{U\}\sqrt{U^2 + \left[4J\cos\left(\tfrac{Kd}{2}\right)\right]^2}. \tag{S50}$$

The eigenfrequencies $\omega_B^{(2)}$ form a single band which may lie above, inside or below the scattering state bands. An example bound state band is plotted as the pink line in Fig. S8 (a) for the case of $U = 4J$. The bound state bandwidth

$$\mathcal{B}_B^{(2)} = \sqrt{U^2 + (4J)^2} - |U|, \tag{S51}$$

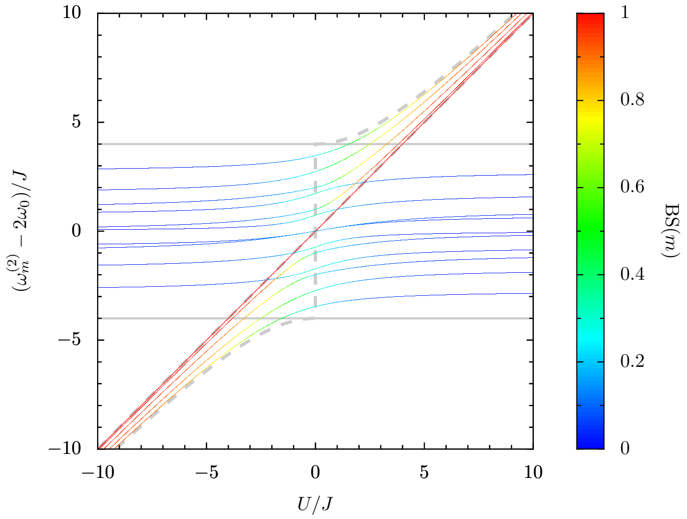

FIG. S9. Chain eigenfrequencies $\omega_m^{(2)}$ in the two excitation sector (as measured from $2\omega_0$) as a function of the interaction strength U, in units of the coupling strength J. The chain has $N = 5$ oscillators. Solid gray lines: region enclosing the scattering states, as defined in the infinite chain limit by Eq. (S41). Dashed gray lines: region enclosing the bound states, as defined in the infinite chain limit by Eq. (S50). Color bar: the bound state ratio $\mathrm{BS}(m)$ of each state m [cf. Eq. (S53)].

is vanishing for large U, as sketched in Fig. S8 (b), as the bound state band tends to become made up of uncoupled doubly excited states. Importantly, this analysis allows us to draw some general conclusions about the form of the band gap $\Delta^{(2)}$ between the bound and scattering bands

$$\Delta^{(2)} = \begin{cases} |U| - 4J, & \text{if} \quad |U| > 4J, \\ 0, & \text{if} \quad |U| \leq 4J, \end{cases} \tag{S52}$$

as shown in Fig. S8 (b), where we plot the extrema of the scattering and bound state bands as a function of the interaction strength U. Of course, for the scattering bands the maxima and minima (thin cyan lines) are independent of U and read $\max\{\omega_{\mathrm{S}}^{(2)}\} = 2\omega_0 + 4J$ and $\min\{\omega_{\mathrm{S}}^{(2)}\} = 2\omega_0 - 4J$. The bound state extrema (thick pink lines) are U dependent, and Fig. S8 (b) highlights that there is a scattering-bound state band gap when $|U| > 4J$.

3. *Numerical diagonalization*

The analytics in the infinite chain limit obtained in the previous parts allowed for some general properties of the system to be obtained. This behavior is seen to indeed hold for chains with a finite number N of oscillators in the chain, as is found from numerical diagonalization (with open boundary conditions) of the $N(N+1)/2 \times N(N+1)/2$ matrix formed from Eq. (S26). The solution of this eigenproblem yields $N(N+1)/2$ eigenfrequencies $\omega_m^{(2)}$, which are associated with the eigenvectors $\psi(m)$. The $N(N+1)/2$-dimensional basis of $\psi(m)$ is comprised of N doubly excited states, like $||2,0,0,...\rangle\rangle$ and $||0,2,0,...\rangle\rangle$, and $N(N-1)/2$ twice singly excited states, like $||1,1,0,...\rangle\rangle$ and $||1,0,1,...\rangle\rangle$. Therefore, one may define the bound state ratio $\mathrm{BS}(m)$ for each state m

$$\mathrm{BS}(m) = \frac{\sum_{n\in\mathrm{bound}} |\psi_n(m)|^2}{\sum_n |\psi_n(m)|^2}, \tag{S53}$$

where $\psi(m) = (\psi_1, \psi_2, \ldots, \psi_{N(N+1)/2})$ is a $N(N+1)/2$-dimensional quantity. The sum in the numerator of Eq. (S53) is taken over the N elements of the eigenfunction associated with doubly excited states, while the sum in the denominator normalizes this quantity by summing over both bound and scattering states, such that $\mathrm{BS}(m) = 1$ for a state wholly comprised of doublons, and $\mathrm{BS}(m) = 0$ for a state not containing any doublons.

In Fig. S9 we plot the eigenfrequencies $\omega_m^{(2)}$ as a function of the interaction strength U for a chain of size $N = 5$ (and so corresponding to a 15-dimensional Hilbert space). The color scale represents the bound state ratio $\mathrm{BS}(m)$ of each state m [cf. Eq. (S53)]. The solid gray lines define a region enclosing the scattering states, as defined in the infinite chain limit by Eq. (S41), while the dashed gray lines mark a region housing the bound states, coming from the infinite chain limit of Eq. (S50). Figure S9

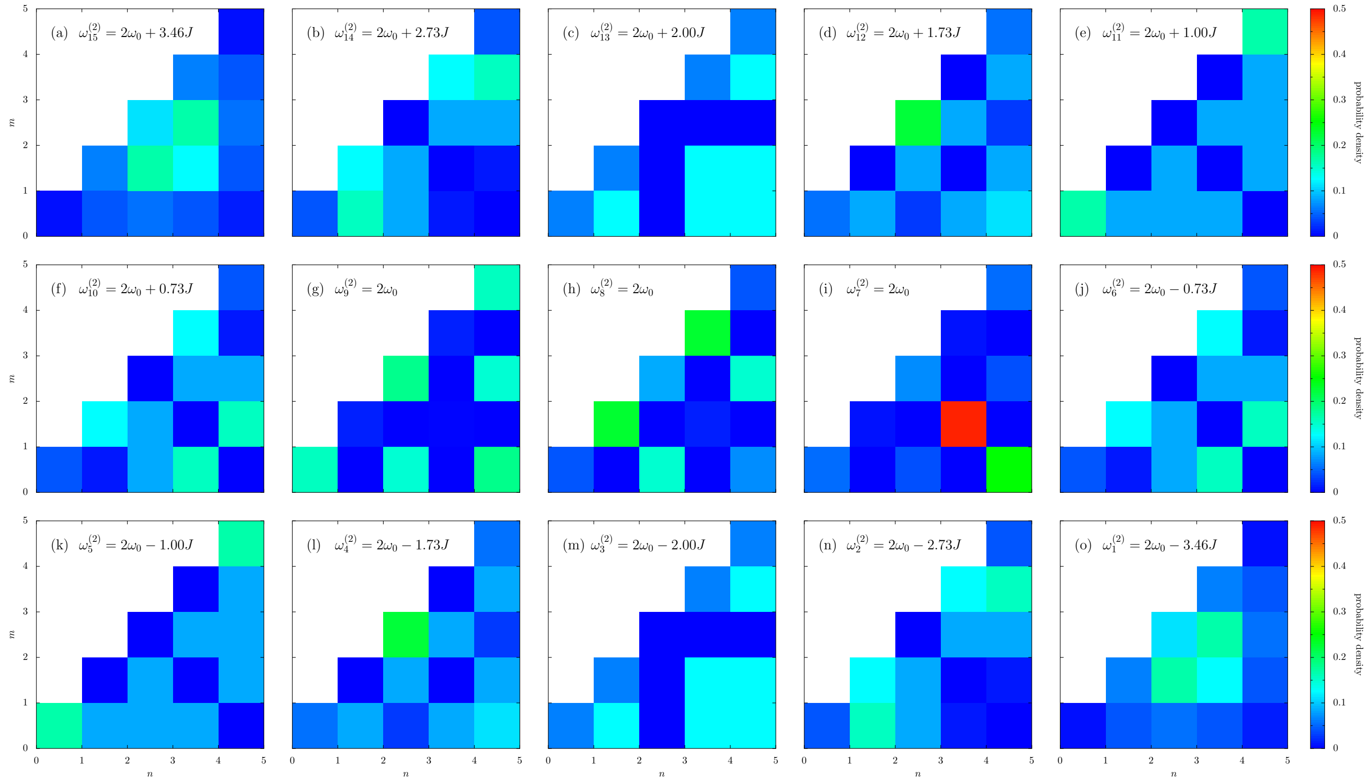

FIG. S10. Probability densities of the chain eigenstates in the two excitation sector. In the figure, the chain has $N = 5$ oscillators and the interaction strength $U = 0$.

generalizes the dimer ($N = 2$) result of Fig. S3, and shows a collection of blue-cyan scattering states (within a band of size $4J$), which are complemented by a handful of red-orange bound states, whose appearance becomes more apparent in the limits of strong attractive and strong repulsive interactions (where they scale linearly with U).

Further characterization of the system can be readily achieved by visualization of the eigenstates. For a system of size N, and with only one excitation, the eigenstate may be best represented on a one-dimensional chain of length N, since the eigenstate is generated from $b_n^\dagger |\text{vac}\rangle$ where $n = 1, 2, ...N$. However, with two excitations the eigenstate should be represented on a two-dimensional shape of base N and height N, as follows from Eq. (S36), since the eigenstate is formed via $b_m^\dagger b_n^\dagger |\text{vac}\rangle$, where both n and m take values from 1 to N. Due to the bosonic symmetry, the simplest representative shape is an isosceles right-angled triangle, where the hypotenuse ($m = n$) represents doubly occupied sites. We present such a representation in Fig. S10 and Fig. S11, for a chain of size $N = 5$, where there are 15 eigenstates. In Fig. S10, we consider the case without interactions ($U = 0$). In this regime, the two excitation eigenfrequencies $\omega_m^{(2)}$ are composed directly from pairs of $\omega_m^{(1)}$, the single excitation eigenfrequencies. As shown in Fig. S10, the fifteen eigenstates correspond to scattering states [cf. Fig. S9] and the probability densities are spread throughout the eigenspace. In Fig. S11, we turn on interactions, and show the results for $U = 5J$. As expected from Fig. S9 at $U = 5J$, the 15 eigenstates are shared between 5 bound states, which are at a higher energy and are displayed in panels (a-e), and 10 lower energy scattering states as shown in panels (f-o). Clearly, the bound states along the upper row of Fig. S11 are highly localized on the doublon hypotenuse, in stark contrast to the scattering states shown in the lower two rows.

4. *Entanglement*

We plot in Fig. S12 two measures of entanglement in the two excitation sector of the regular chain: the purity ζ_m of each state in panel (a), and the the von Neumann entropy S_m in panel (b). The results are for a chain of $N = 8$ oscillators, so the Hilbert space is 36-dimensional. The purity plot in Fig. S12 (a) clearly identifies the bound states (red-orange on this color scale) which are characteristically less mixed, and the scattering states (blue-green on this color scale) which are highly mixed due to their extended nature. The von Neumann entropy plot in Fig. S12 (b) confirms the distinction between bound and scattering states.

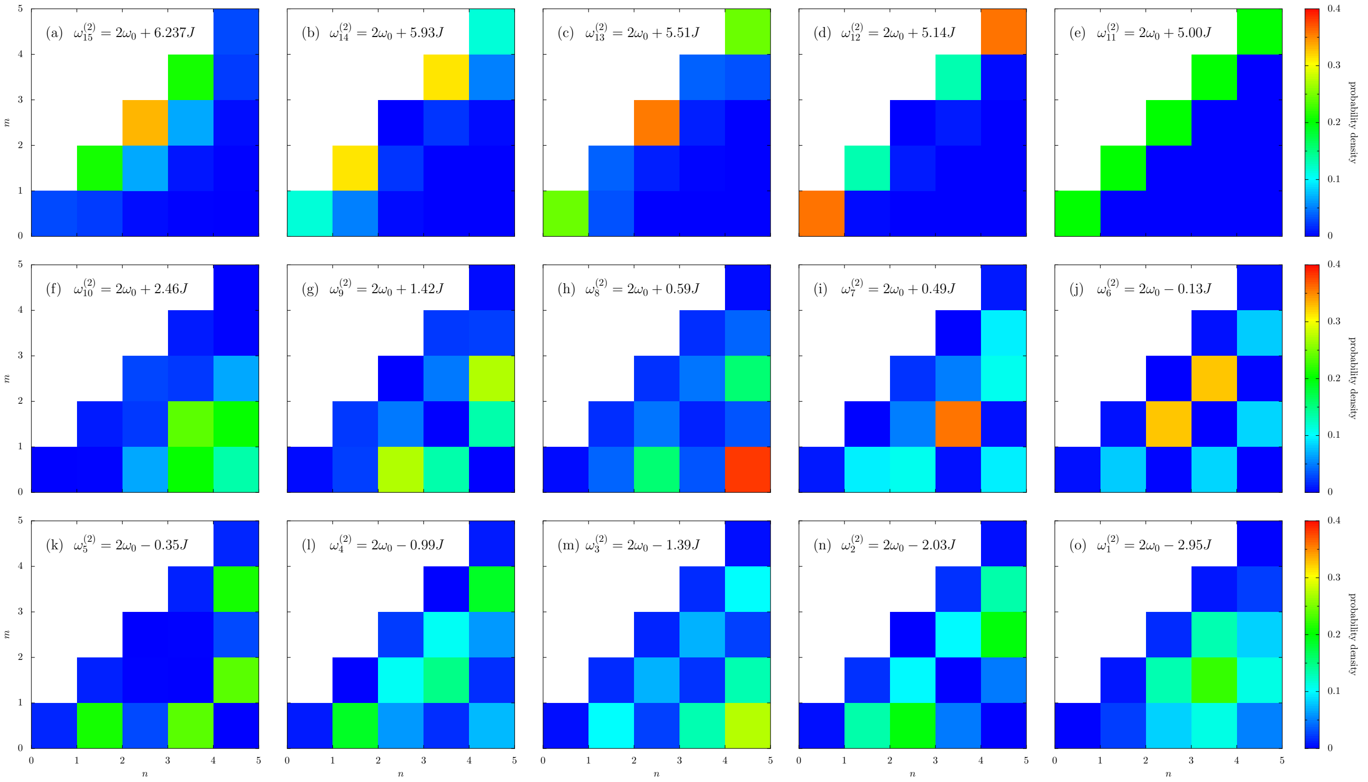

FIG. S11. Probability densities of the chain eigenstates in the two excitation sector. In the figure, the chain has $N = 5$ oscillators and the interaction strength $U = 5J$.

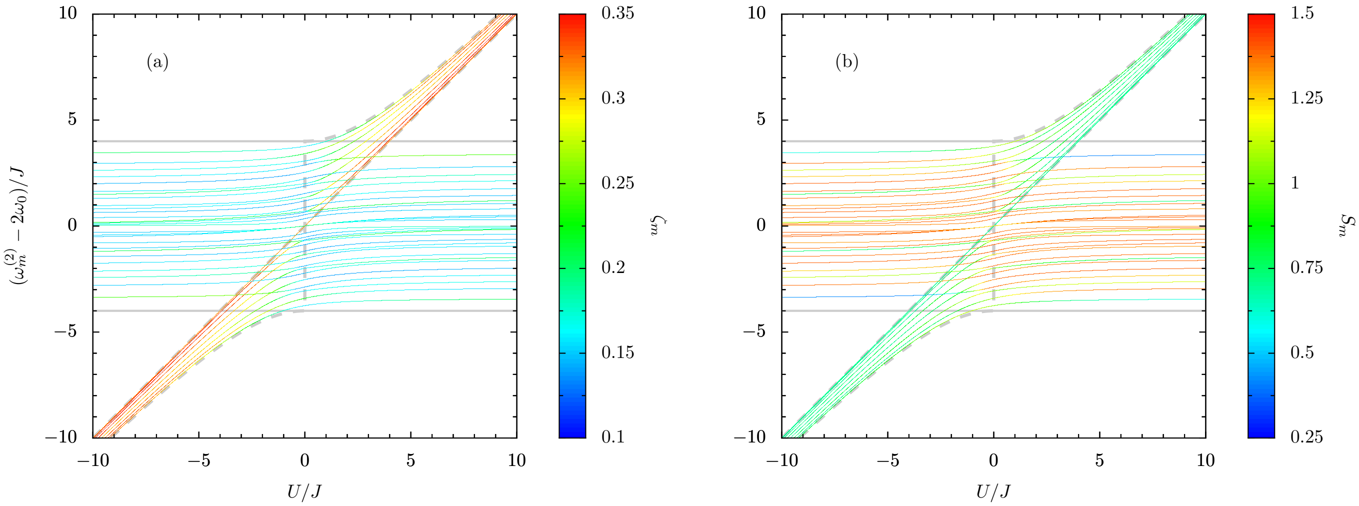

FIG. S12. Panel (a): purity ζ_m of the chain states in the two excitation sector, as a function of the eigenfrequencies $\omega_m^{(2)}$ (as measured from $2\omega_0$), and the interaction strength U, in units of the coupling strength J. The chain is of size $N = 8$. Panel (b): the von Neumann entropy S_m. Solid gray lines: region enclosing the scattering states, as defined in the infinite chain limit by Eq. (S41). Dashed gray lines: region enclosing the bound states, as defined in the infinite chain limit by Eq. (S50).

IV. BOSE-HUBBARD DIMERIZED CHAIN

In this section, we provide some supplementary results on the Bose-Hubbard dimerized chain model, as described by Eq. (1) in the main text and sketched in Fig. S13. In particular, we recap some information about the one excitation sector in Sec. IV A, and provide some results in the two excitation sector in Sec. IV B.

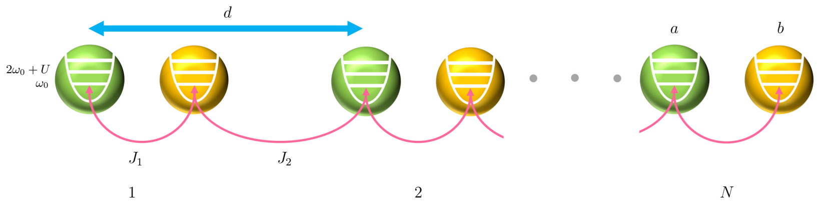

FIG. S13. A sketch of the Bose-Hubbard dimerized chain of N dimers, where the effective lattice constant is d. Each dimer contains two oscillators (of type a and b) and both are of resonance frequency ω_0. The two nearest-neighbor coupling constants $-J_1, -J_2 < 0$ are attractive, and the on-site interaction U may be repulsive ($U > 0$) or attractive ($U < 0$).

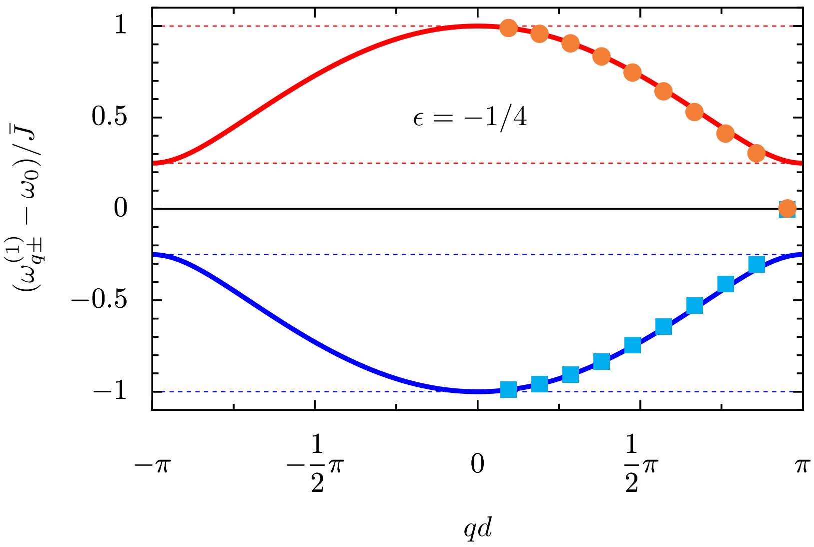

FIG. S14. Eigenfrequencies $\omega^{(1)}_{q\pm}$ of the infinite dimerized chain in the one excitation sector (as measured from ω_0), in units of the coupling strength $\bar{J}$, as a function of the wavevector q [cf. Eq. (S61)]. Orange circles and cyan squares: eigenfrequencies $\omega^{(1)}_{m\pm}$ for a finite chain of $N = 10$ dimers. Dashed lines: guides for the eye. In the figure, $\epsilon = -1/4$.

A. One excitation subspace

In the one excitation subspace $\mathcal{H}_1$ the interaction term is inconsequential, and so the Hamiltonian of Eq. (1) in the main text reduces to the quadratic form

$$\mathcal{H}_1 = \omega_0 \sum_{n=1}^{N} \left(a_n^\dagger a_n + b_n^\dagger b_n\right) - J_1 \sum_{n=1}^{N} \left(b_n^\dagger a_n + a_n^\dagger b_n\right) - J_2 \sum_{n=1}^{N} \left(b_n^\dagger a_{n+1} + a_{n+1}^\dagger b_n\right), \tag{S54}$$

where the creation (annihilation) operators $a_n^\dagger$ and $b_n^\dagger$ (a_n and b_n) create (destroy) an excitation on oscillator a and b in dimer n in the chain. All oscillators are of resonance frequency ω_0, and the two nearest-neighbor coupling constants $-J_1, -J_2 < 0$ are attractive [cf. Fig. S13]. We analyze this Hamiltonian in the infinite ($N \gg 1$) chain limit in Part IV A 1, and for a general finite chain in Part IV A 2.

1. *Infinite chain*

We use periodic boundary conditions when working in the long chain limit of $N \gg 1$ dimers, and employ the pair of exponential Fourier transforms

$$a_n = \frac{1}{\sqrt{N}} \sum_q \mathrm{e}^{inqd} a_q, \quad b_n = \frac{1}{\sqrt{N}} \sum_q \mathrm{e}^{inqd} b_q, \tag{S55}$$

where the quasi-momentum $q = 2\pi m/Nd$, where the integer $m \in [-N/2, +N/2]$. The momentum space creation (annihilation) operators $a_q^\dagger$ and $b_q^\dagger$ (a_q and b_q) create (destroy) an excitation with wavenumber q on an oscillator of type a or b, which are

sketched in green and yellow respectively in Fig. S13. This transformation yields with Eq. (S54) the Hamiltonian

$$\mathcal{H}_1 = \sum_q \hat{\psi}_q^\dagger \mathcal{H}_q \hat{\psi}_q, \quad \hat{\psi}_k = \begin{pmatrix} a_q \\ b_q \end{pmatrix}, \tag{S56}$$

where the matrix Bloch Hamiltonian $\mathcal{H}_q$ reads

$$\mathcal{H}_q = \omega_0 \mathcal{I}_2 - \begin{pmatrix} 0 & Q\mathrm{e}^{-\mathrm{i}\theta_q} \\ Q\mathrm{e}^{\mathrm{i}\theta_q} & 0 \end{pmatrix}, \tag{S57}$$

where the 2×2 identity matrix is $\mathcal{I}_2$, and we have introduced the key quantity

$$Q\mathrm{e}^{\mathrm{i}\theta_q} = J_1 + J_2 \mathrm{e}^{\mathrm{i}qd}. \tag{S58}$$

The diagonal form of Eq. (S56) readily follows as

$$\mathcal{H}_1 = \sum_q \left(\omega_{q+}^{(1)} \beta_{q+}^\dagger \beta_{q+} + \omega_{q-}^{(1)} \beta_{q-}^\dagger \beta_{q-} \right), \tag{S59}$$

where the bosonic Bogoliubov operators $\beta_{q\pm}$ are given by

$$\beta_{q\pm} = \frac{1}{\sqrt{2}} \left(a_q \mp \mathrm{e}^{\mathrm{i}\theta_q} b_q \right), \tag{S60}$$

and where the eigenfrequencies $\omega_{q\pm}^{(1)}$ read

$$\omega_{q\pm}^{(1)} = \omega_0 \pm \sqrt{J_1^2 + J_2^2 + 2J_1 J_2 \cos(qd)}. \tag{S61}$$

We plot in Fig. S14 the eigenfrequencies $\omega_{q\pm}^{(1)}$ in the first Brillouin zone for the case where $J_2 = (5/3)J_1$, with the $+$ $(-)$ band shown by the solid red (blue) line. The two-band spectrum of Eq. (S61) displays a minimum gap at the edge of the Brillouin zone $q = \pm\pi/d$, given by

$$\Delta^{(1)} = 2|J_1 - J_2|, \tag{S62}$$

which only closes when $J_1 = J_2$ at the quasimomentum $q = \pm\pi/d$. As can be seen from Fig. S14, both bands are of equal bandwidth

$$\mathcal{B}_\pm^{(1)} = \begin{cases} 2J_2, & \text{if } J_2 < J_1, \\ 2J_1, & \text{if } J_2 > J_1, \end{cases} \tag{S63}$$

as is alluded to by the dashed lines in the figure. Instead of J_1 and J_2, it is useful to parameterize the dimerized system with the coupling strength

$$\bar{J} = J_1 + J_2, \tag{S64}$$

and the dimerization parameter

$$\epsilon = \frac{J_1 - J_2}{\bar{J}}, \tag{S65}$$

so that $J_1 = \bar{J}(1+\epsilon)/2$ and $J_2 = \bar{J}(1-\epsilon)/2$. The Bloch states of the system arise from Eq. (S57), and read

$$|\psi_{q\pm}\rangle = \frac{1}{\sqrt{2}} \begin{pmatrix} 1 \\ \mp\mathrm{e}^{\mathrm{i}\theta_q} \end{pmatrix}. \tag{S66}$$

The celebrated topological invariant for such single excitation, one-dimensional systems is the Zak phase [7]. It is defined by the following integral over the first Brillouin zone,

$$\phi_{\mathrm{Zak}}^\pm = \int_{\mathrm{BZ}} \mathrm{i}\langle \psi_{q\pm} | \partial_q | \psi_{q\pm} \rangle \mathrm{d}q. \tag{S67}$$

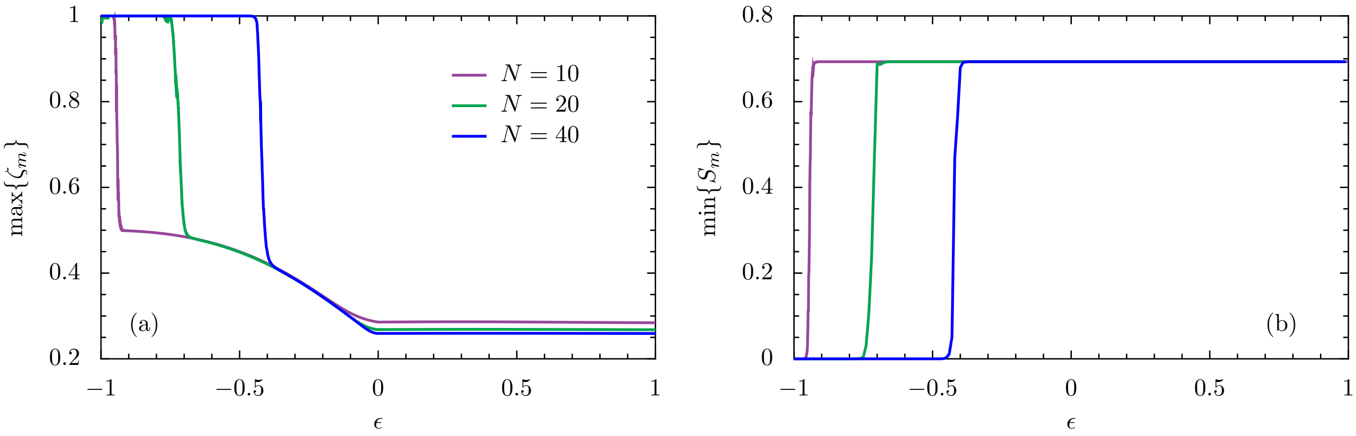

FIG. S15. Panel (a): Maximum of the purity ζ_m of the dimerized chain states (labelled by m) in the one excitation sector, as a function of the dimerization ϵ. Panel (b): minimum of the von Neumann entropy S_m. The number of dimers N in the chain is given by the legend.

Substituting Eq. (S66) into Eq. (S67) yields the well-known result

$$\phi_{\text{Zak}}^{\pm} = \begin{cases} \pi, & \text{if } \epsilon < 0, \\ 0, & \text{if } \epsilon \geq 0, \end{cases} \tag{S68}$$

which implies a topologically nontrivial regime when $J_2 > J_1$ (associated with the Zak phase of π), and a topologically trivial regime when $J_2 \leq J_1$ (linked to a Zak phase of 0). The bulk-edge correspondence guarantees that the topologically nontrivial phase is linked to the existence of edge states [8]. An example of this correspondence is shown in Fig. S14, where the orange circles and cyan squares represent the eigenfrequencies $\omega_{m\pm}^{(1)}$ as calculated numerically for a finite chain of $N = 10$ dimers. Most notably, while the continuum calculation (solid lines) has missed the two topologically protected edge state residing at ω_0, these edge states are implied by the continuum calculation via the Zak phase as encapsulated by Eq. (S68).

2. *Finite chain*

In order to study a finite chain of N dimers (and so $2N$ oscillators in total) one must diagonalize the $2N \times 2N$ matrix formed from Eq. (S54). The eigenvalues of this matrix yield the the eigenfrequencies $\omega_{m\pm}^{(1)}$, as indexed by the integer m. The eigenfunctions of this matrix give information about the nature of each eigenstate, for example the degree of localization is quantified via the participation ratio $\text{PR}(m)$ of each state m, as defined by [9, 10]

$$\text{PR}(m) = \frac{\left(\sum_n |\psi_n(m)|^2\right)^2}{\sum_n |\psi_n(m)|^4}, \tag{S69}$$

where $\psi(m) = (\psi_1, \psi_2, \ldots, \psi_{2N})$ is a $2N$-dimensional eigenfunction arising from diagonalizing the $2N \times 2N$ Hamiltonian for a finite chain of N dimers. The participation ratio $\text{PR}(m)$ distinguishes scattering states, which scale like $\text{PR}(m) \propto N^1$, from bound states, which scale like $\text{PR}(m) \propto N^0$. The evolution of the system from topologically nontrivial to topologically trivial is shown in Fig. 2 of the main text, which presents the results of diagonalizing a finite system of $N = 10$ dimers, as a function of the dimerization parameter ϵ. Figure 2 of the main text clearly shows for $\epsilon < 0$ the existence of topological midgap states at ω_0 (red-orange on this color scale), which are lost for $\epsilon \geq 0$ as a direct consequence of entering a topologically trivial phase full of scattering states (blue-green on this color scale), as predicted by the topological invariant of Eq. (S68). The utility of the participation ratio to distinguish different types of state, be they topological or non-topological, is exemplified in Refs. [11, 12].

3. *Entanglement*

The topology of the Su-Schrieffer-Heeger model is captured via some standard entanglement properties, as displayed in Fig. S15. In Fig. S15 (a) we plot the maximum value of the purity ζ_m after considering all states of a certain dimerization ϵ. Notably in the topological phase $\epsilon < 0$ this quantity is high, due to the presence of largely pure edge states, while in the non-topological phase $\epsilon \geq 0$ this quantity is low, since there are only highly mixed scattering states. In Fig. S15 (a) we show

results for increasingly long chains with $N = \{10, 20, 40\}$ dimers in purple, green and blue lines respectively, which shows the intuitive trend of the transition from a system with high purity states to all low purity states occurring increasingly close to $\epsilon = 0$ with increasing chain length. A calculation of the minimum of von Neumann entropy in the system for each dimerization ϵ is shown in Fig. S15 (b) and fully complements the features found in panel (a).

B. Two excitation subspace

In the two excitation subspace, the Bethe ansatz solution can be employed in a similar manner as for the regular chain, as shown by Gorlach and Poddubny in Ref. [13]. Let us substitute the eigenstate

$$|\psi\rangle = \sum_{m,n=1}^{N} A_{m,n}\, a_m^\dagger a_n^\dagger |\text{vac}\rangle + \sum_{m,n=1}^{N} B_{m,n}\, b_m^\dagger b_n^\dagger |\text{vac}\rangle + \sum_{m,n=1}^{N} C_{m,n}\, a_m^\dagger b_n^\dagger |\text{vac}\rangle + \sum_{m,n=1}^{N} D_{m,n}\, b_m^\dagger a_n^\dagger |\text{vac}\rangle, \tag{S70}$$

into the Schrödinger equation $\hat{H}|\psi\rangle = \omega^{(2)}|\psi\rangle$, where $\hat{H}$ is defined in Eq. (1) of the main text and $\omega^{(2)}$ are the two-particle eigenvalues. We arrive at four coupled recurrence equations for the weighting coefficients $A_{m,n}$, $B_{m,n}$, $C_{m,n}$ and $D_{m,n}$

$$-J_1 (C_{m,n} + D_{m,n}) - J_2 (C_{m,n-1} + D_{m-1,n}) = \left(\omega^{(2)} - 2\omega_0 - U\delta_{m,n}\right) A_{m,n}, \tag{S71}$$

$$-J_1 (C_{m,n} + D_{m,n}) - J_2 (C_{m+1,n} + D_{m,n+1}) = \left(\omega^{(2)} - 2\omega_0 - U\delta_{m,n}\right) B_{m,n}, \tag{S72}$$

$$-J_1 (A_{m,n} + B_{m,n}) - J_2 (A_{m,n+1} + B_{m-1,n}) = \left(\omega^{(2)} - 2\omega_0\right) C_{m,n}, \tag{S73}$$

$$-J_1 (A_{m,n} + B_{m,n}) - J_2 (A_{m+1,n} + B_{m,n-1}) = \left(\omega^{(2)} - 2\omega_0\right) D_{m,n}. \tag{S74}$$

The following ansatz describes scattering solutions

$$\begin{pmatrix} A_{m,n} \\ B_{m,n} \\ C_{m,n} \\ D_{m,n} \end{pmatrix} = \begin{pmatrix} \alpha \\ \beta \\ \gamma \\ \delta \end{pmatrix} \mathrm{e}^{\mathrm{i}k_1 dm} \mathrm{e}^{\mathrm{i}k_2 dn}, \tag{S75}$$

where k_1 and k_2 are the wavevectors associated with the two particle problem, and $\{\alpha, \beta, \gamma, \delta\}$ are unknowns to be found. Together, Eq. (S71) and Eq. (S75) imply the four-dimensional matrix equation

$$\begin{pmatrix} U\delta_{m,n} & 0 & -J_1 - J_2\mathrm{e}^{-\mathrm{i}k_2 d} & -J_1 - J_2\mathrm{e}^{-\mathrm{i}k_1 d} \\ 0 & U\delta_{m,n} & -J_1 - J_2\mathrm{e}^{\mathrm{i}k_1 d} & -J_1 - J_2\mathrm{e}^{\mathrm{i}k_2 d} \\ -J_1 - J_2\mathrm{e}^{\mathrm{i}k_2 d} & -J_1 - J_2\mathrm{e}^{-\mathrm{i}k_1 d} & 0 & 0 \\ -J_1 - J_2\mathrm{e}^{\mathrm{i}k_1 d} & -J_1 - J_2\mathrm{e}^{-\mathrm{i}k_2 d} & 0 & 0 \end{pmatrix} \begin{pmatrix} \alpha \\ \beta \\ \gamma \\ \delta \end{pmatrix} = \omega^{(2)} \begin{pmatrix} \alpha \\ \beta \\ \gamma \\ \delta \end{pmatrix}. \tag{S76}$$

The resulting secular equation is bi-quadratic and suggests the four scattering band eigenfrequencies with $m \neq n$ (or $U = 0$)

$$\omega_{\pm\pm}^{(2)} = \pm\sqrt{J_1^2 + J_2^2 + 2J_1J_2\cos\left(Kd/2 + kd\right)} \pm \sqrt{J_1^2 + J_2^2 + 2J_1J_2\cos\left(Kd/2 - kd\right)}, \tag{S77}$$

where the center-of-mass wavevector $K = k_1 + k_2$ and relative motion wavevector $k = (k_2 - k_1)/2$, that is $k_1 = K/2 + k$ and $k_2 = K/2 - k$. This expression is intuitive since it corresponds to the sum of contributions from two non-interacting excitations with quasimomentum k_1 and k_2 [cf. Eq. (S61)].

Using Eq. (S77), we plot the scattering bands $\omega_{\pm\pm}^{(2)}$ in Fig. S16 for increasingly large dimerization ϵ across the row of panels. As in the single excitation sector, increasingly large dimerization leads to more widely separated bands. The central $\omega_{+-}^{(2)}$ and $\omega_{-+}^{(2)}$ bands (green and orange in the figure) are always connected, such that it in any calculation for a finite chain only three scattering bands would appear (see Fig. 3 in the main text for example). The exact continuum expression given in Eq. (S77) are

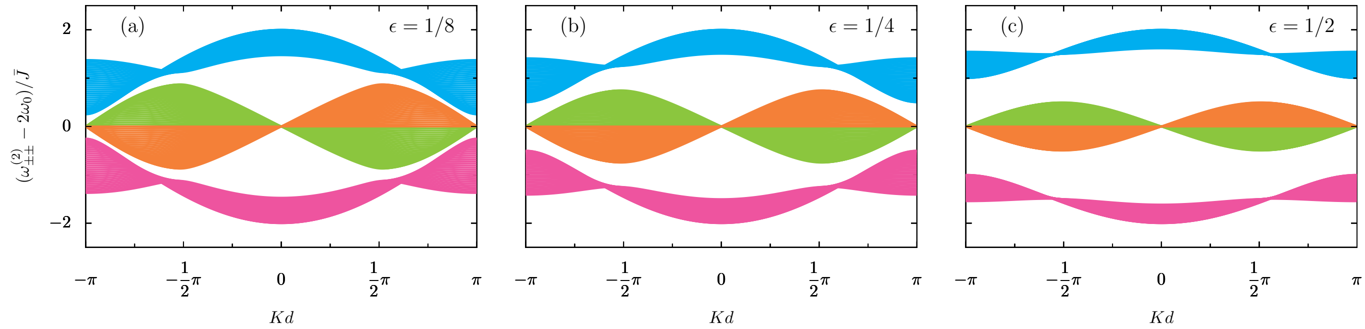

FIG. S16. Eigenfrequencies $\omega^{(2)}_{\pm\pm}$ of the scattering bands belonging to an infinitely long dimerized chain in the two excitation sector (as measured from $2\omega_0$), in units of the coupling strength $\bar{J}$, as a function of the center-of-mass wavevector K [cf. Eq. (S77)]. The dimerization ϵ increases across the row of panels. Blue bands: $\omega^{(2)}_{++}$. Green bands: $\omega^{(2)}_{+-}$. Orange bands: $\omega^{(2)}_{-+}$. Pink bands: $\omega^{(2)}_{--}$.

used throughout the main text to demarcate the energy sectors associated with scattering bands in the main text (usually with dashed gray lines in the main text figures).

---

[1] M. A. Nielsen and I. L. Chuang, *Quantum Computation and Quantum Information*, (Cambridge University Press, Cambridge, 2000).
[2] M. Schlosshauer, *Decoherence and the Quantum-To-Classical Transition*, (Springer, Berlin, 2007).
[3] H. Li and F. D. M. Haldane, Entanglement spectrum as a generalization of entanglement entropy: Identification of topological order in non-Abelian fractional quantum Hall effect states, Phys. Rev. Lett. **101**, 010504 (2008).
[4] N. Regnault, Entanglement spectroscopy and its application to the quantum Hall effects, Lecture Notes Les Houches Summer School **103**, 165 (2017).
[5] M. Valiente and D. Petrosyan, Two-particle states in the Hubbard model, J. Phys. B: At. Mol. Opt. Phys. **41**, 161002 (2008).
[6] M. Valiente and D. Petrosyan, Scattering resonances and two-particle bound states of the extended Hubbard model, J. Phys. B: At. Mol. Opt. Phys. **42**, 121001 (2009).
[7] J. Zak, Berry's phase for energy bands in solids, Phys. Rev. Lett. **62**, 2747 (1989).
[8] J. K. Asboth, L. Oroszlany, and A. Palyi, *A Short Course on Topological Insulators* (Springer, Heidelberg, 2016).
[9] R. J. Bell and P. Dean, Atomic vibrations in vitreous silica, Discuss. Faraday Soc. **50**, 55 (1970).
[10] D. J. Thouless, Electrons in disordered systems and the theory of localization, Phys. Rep. **13**, 93 (1974).
[11] C. A. Downing, T. J. Sturges, G. Weick, M. Stobińska, and L. Martín-Moreno, Topological phases of polaritons in a cavity waveguide, Phys. Rev. Lett. **123**, 217401 (2019).
[12] C. A. Downing and L. Martín-Moreno, Polaritonic Tamm states induced by cavity photons, Nanophotonics **10**, 513 (2021).
[13] M. A. Gorlach, and A. N. Poddubny, Topological edge states of bound photon pairs, Phys. Rev. A **95**, 053866 (2017).
